# Ivermectin Attenuates Methotrexate-Induced Liver Fibrosis by Reducing TGF-β and Syndecan-1 Expression

**DOI:** 10.3390/medicina61061036

**Published:** 2025-06-04

**Authors:** Cengiz Dibekoğlu, Kubilay Kemertaş, Hatice Aygun, Oytun Erbaş

**Affiliations:** 1 Department of General Surgery, Demiroğlu Bilim University, 34394 Istanbul, Turkey; cdibekoglu@gmail.com; 2 Department of General Surgery, Florence Nightingale Hospital, 34394 Istanbul, Turkey; drkemertas@gmail.com; 3 Department of Physiology, Faculty of Medicine, Tokat Gaziosmanpaşa University, 60250 Tokat, Turkey; 4 Faculty of Medicine, BAMER, Biruni University, 34015 Istanbul, Turkey; oytunerbas2012@gmail.com

**Keywords:** ivermectin, methotrexate, hepatotoxicity, liver fibrosis, oxidative stress, TGF-β, syndecan-1

## Abstract

*Background and Objectives:* Methotrexate (MTX) is widely used in clinical settings but is often associated with hepatotoxic side effects, including oxidative stress, inflammation, and fibrosis. Novel therapeutic strategies are needed to mitigate MTX-induced liver injury. This study aimed to evaluate the hepatoprotective effects of ivermectin in a rat model of MTX-induced hepatotoxicity. *Materials and Methods:* Thirty male Wistar albino rats were randomly divided into three groups (n = 10 per group): control (saline only), MTX (single intraperitoneal dose of 20 mg/kg MTX), and MTX + ivermectin (20 mg/kg MTX + 0.5 mg/kg/day ivermectin for 10 days). At the end of the experiment, blood and liver tissues were collected for histopathological and biochemical evaluation, including ALT, malondialdehyde (MDA), TGF-β, and syndecan-1 levels. *Results:* MTX administration significantly increased plasma and hepatic MDA, TGF-β, syndecan-1, and ALT levels, alongside histological evidence of necrosis, fibrosis, and inflammatory infiltration (*p* < 0.001 vs. control). Ivermectin treatment significantly attenuated these alterations, with reductions in MDA (both plasma and liver), TGF-β, syndecan-1, and ALT levels (*p* < 0.05–0.001 vs. MTX). Histological scoring also revealed improved liver architecture and decreased necrosis, fibrosis, and leukocyte infiltration. *Conclusions:* Ivermectin demonstrates a strong hepatoprotective effect against MTX-induced liver injury, likely through antioxidant, anti-inflammatory, antifibrotic, and endothelial-protective mechanisms. These findings support the repurposing potential of ivermectin in mitigating drug-induced hepatic damage.

## 1. Introduction

Methotrexate (MTX) is a folate antagonist that is widely used in the treatment of malignancies and autoimmune diseases due to its antiproliferative and immunosuppressive effects (Weinblatt, 2013) [1]. However, its clinical utility is often limited by dose-dependent hepatotoxicity. MTX-induced liver injury is multifactorial, involving oxidative stress, mitochondrial dysfunction, inflammation, endothelial injury, and progressive fibrogenesis [2,3].

Accumulating evidence suggests that MTX increases reactive oxygen species (ROS) and lipid peroxidation, triggering hepatocyte apoptosis and necrosis [2,3]. Concomitantly, MTX promotes the expression of profibrotic cytokines such as transforming growth factor-β (TGF-β), which activates hepatic stellate cells (HSCs), initiating collagen deposition and tissue remodeling [4,5,6]. In parallel, disruption of the endothelial glycocalyx—reflected by increased shedding of syndecan-1 (SDC1), a heparan sulfate proteoglycan—contributes to hepatic microvascular dysfunction [7]. Despite these mechanistic insights, most hepatoprotective strategies target single pathogenic pathways and often overlook extracellular matrix dynamics and endothelial markers.

Ivermectin is a broad-spectrum antiparasitic agent originally derived from *Streptomyces avermitilis* and approved by the United States Food and Drug Administration (FDA) [8,9]. Its primary mechanism of action involves high-affinity binding to glutamate-gated chloride channels (GluCls), predominantly expressed in invertebrate nerve and muscle cells, resulting in chloride influx, neuronal hyperpolarization, and paralysis [10,11]. The absence of these channels in mammalian systems in the same configuration underlies ivermectin’s favorable safety profile at therapeutic doses [12,13]. Beyond its antiparasitic properties, recent studies have highlighted its anti-inflammatory, antioxidant, and antifibrotic effects, which are believed to be mediated via modulation of key signaling pathways such as nuclear factor kappa B (NF-κB), nuclear factor erythroid 2-related factor 2 (Nrf2), and transforming growth factor-beta/Smad (TGF-β/Smad), as well as interactions with ligand-gated ion channels and purinergic P2X4 receptors [13,14,15,16]. These pleiotropic effects have prompted interest in repurposing ivermectin for the treatment of non-infectious inflammatory conditions, including neurodegenerative disorders, malignancies, and organ-specific toxicities, such as hepatotoxicity [9,15,17]. Nonetheless, the potential of ivermectin to prevent or mitigate methotrexate (MTX)-induced hepatic injury remains largely unexplored.

The present study aims to investigate, for the first time, the hepatoprotective effects of ivermectin in an experimental rat model of MTX-induced hepatotoxicity. In addition to conventional biomarkers (alanine aminotransferase [ALT], malondialdehyde [MDA], TGF-β), we evaluated syndecan-1 as a novel marker of hepatic extracellular matrix disruption and endothelial injury. By integrating biochemical, molecular, and histopathological parameters, this study provides a comprehensive analysis of ivermectin’s potential to modulate oxidative stress, inflammation, fibrosis, and endothelial damage in MTX-induced liver injury.

## 2. Materials and Methods

### 2.1. Animals

This study was conducted on 30 healthy male Wistar albino rats (10–12 weeks old, 150–200 g). All animal procedures were approved by the Animal Experiments Local Ethics Committee of Science University (Approval No.: 0223202112) and conducted in accordance with the National Institutes of Health (NIH, Bethesda, MD, USA) guidelines and the ARRIVE (Animal Research: Reporting of In Vivo Experiments) guidelines.

Animals were obtained from the Experimental Animal Research Center of Science University and acclimatized for one week prior to experimentation. They were housed in pairs under standardized conditions (22 ± 2 °C, 12-h light/dark cycle), with free access to standard chow and tap water. General health, behavior, and potential signs of distress were monitored daily.

### 2.2. Experimental Protocol

A total of 30 male Wistar albino rats were randomly divided into three experimental groups, each containing 10 animals:

Group 1—Control Group: Healthy rats that received 0.9% NaCl solution (2 mL/kg/day, intraperitoneally) for 10 consecutive days. No methotrexate or ivermectin was administered to this group.

Group 2—MTX Group: Rats received a single intraperitoneal injection of methotrexate (20 mg/kg, i.p.) to induce hepatotoxicity, followed by daily administration of 0.9% NaCl solution (2 mL/kg/day, i.p.) for 10 days.

Group 3—MTX + Ivermectin Group: Rats were administered a single dose of methotrexate (20 mg/kg, i.p.) and subsequently treated with ivermectin (0.5 mg/kg/day, i.p.) for 10 consecutive days.

**Figure 1:**
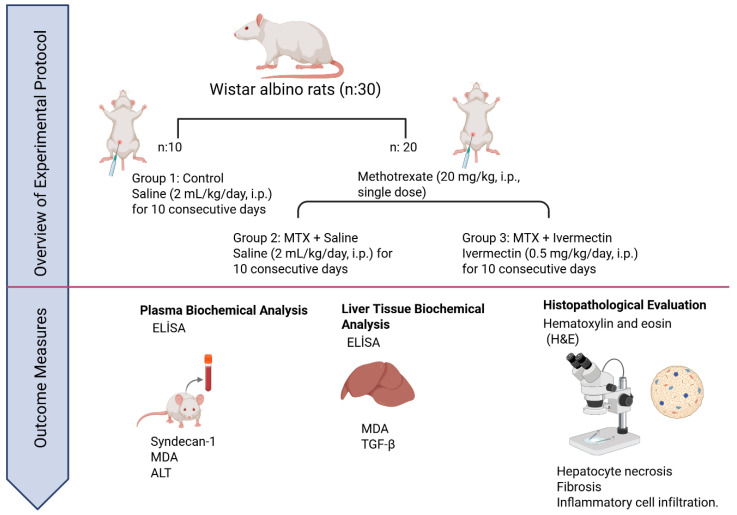
Schematic representation of the experimental protocol and outcome assessments: Thirty male Wistar albino rats were randomly divided into three groups (n = 10 per group). Group 1 (control) received 0.9% saline (2 mL/kg/day, intraperitoneally [i.p.]) for 10 consecutive days. Group 2 received a single dose of methotrexate (MTX, 20 mg/kg, i.p.), followed by saline (2 mL/kg/day, i.p.) for 10 days. Group 3 received MTX as in Group 2, followed by ivermectin (0.5 mg/kg/day, i.p.) for 10 days. Blood and liver tissue samples were collected for biochemical analyses, including plasma syndecan-1 (SDC1), malondialdehyde (MDA), and alanine aminotransferase (ALT), as well as hepatic MDA and transforming growth factor-beta (TGF-β) levels, using ELISA. Histopathological evaluation of liver sections was performed with hematoxylin and eosin (H&E) staining to assess hepatocyte necrosis, fibrosis, and inflammatory cell infiltration.

At the end of the treatment period, all rats were anesthetized with ketamine (90 mg/kg, i.p.) and xylazine (10 mg/kg, i.p.), and then euthanized via decapitation. Blood was collected via cardiac puncture, and livers were immediately excised under sterile conditions for histopathological and biochemical analyses. All procedures were performed at consistent times to minimize circadian variation (Figure 1).

### 2.3. Histopathological Evaluation

Liver tissues were fixed in 10% neutral-buffered formalin for at least 48 h. Following dehydration, the tissues were embedded in paraffin, sectioned into 4 µm slices using a rotary microtome, and mounted on glass slides. Sections were stained with hematoxylin and eosin (H&E) to assess their general histological architecture. Microscopic evaluation was conducted using an Olympus BX51 light microscope (Olympus, Tokyo, Japan) equipped with a C-5050 digital imaging system (Azure Biosystems, Dublin, CA, USA).

Histological damage was scored using a semi-quantitative scale adapted from Lobenhofer et al. (2006) [18]. A blinded pathologist performed the scoring based on hepatocyte necrosis, fibrosis, and inflammatory cell infiltration [18]. Lesions were graded on a five-point severity scale:

0: Absent;
1: Minimal (<5% involvement);
2: Mild (5–25%);
3: Moderate (26–50%);
4: Marked (>50%).

### 2.4. Biochemical Evaluation

#### 2.4.1. Plasma Analysis

At the end of the experimental period, all animals were anesthetized with ketamine (90 mg/kg, i.p.) and xylazine (10 mg/kg, i.p.), followed by euthanasia via decapitation using a guillotine. Blood samples were collected via cardiac puncture into sterile tubes and allowed to clot at room temperature for 30 min. The samples were then centrifuged at 3000 rpm for 10 min to separate the plasma. The resulting supernatant was carefully collected and stored at −20 °C until biochemical analysis.

Plasma levels of syndecan-1 (ng/mL), malondialdehyde (MDA, nM), and alanine aminotransferase (ALT, IU/L) were quantified using commercially available enzyme-linked immunosorbent assay (ELISA) kits (R&D Systems, Minneapolis, MN, USA), according to the manufacturer’s instructions. All measurements were performed in duplicate to ensure analytical accuracy and reproducibility.

##### Plasma Syndecan-1 Measurement

Plasma levels of syndecan-1, an endothelial glycocalyx marker associated with tissue injury and inflammation, were measured using a rat-specific ELISA kit (Biosciences, Dural, NSW, Australia). Samples were diluted 1:2 and analyzed in duplicate according to the manufacturer’s instructions. The absorbance was read using a microplate spectrophotometer at 450 nm.

##### Plasma ALT Determination

ALT activity, a key biomarker of hepatic cell damage, was measured in serum samples using a commercial ELISA kit (USCN Life Science Inc., Houston, TX, USA). All samples were run in duplicate, and ALT concentrations were calculated based on the provided standard curve.

##### Plasma Lipid Peroxidation

The extent of lipid peroxidation in plasma was quantified by assessing MDA levels using the thiobarbituric acid-reactive substances (TBARS) assay. Equal volumes of trichloroacetic acid and TBA reagent were mixed with the plasma, heated at 100 °C for 60 min, cooled on ice, and centrifuged at 3000 rpm for 20 min. The absorbance of the resulting supernatant was measured at 535 nm, and MDA concentrations were calculated using a standard curve prepared with tetraethoxypropane.

#### 2.4.2. Liver Tissue Biochemical Analysis

Immediately after euthanasia, liver samples were rapidly harvested, weighed, and frozen at −20 °C. For biochemical assays, tissue homogenates were prepared by homogenizing liver samples in phosphate-buffered saline (PBS; pH 7.4) at a 1:5 weight-to-volume ratio. Homogenates were centrifuged at 5000× *g* for 15 min, and the supernatants were used for the following analyses: TGF-β (pg/g tissue) and MDA (nmol/g tissue).

Protein concentrations in homogenates were determined by the Bradford method, using bovine serum albumin as a standard [19].

##### Liver TGF-β Measurement

TGF-β, a profibrotic cytokine implicated in hepatic inflammation and fibrosis, was measured using a rat-specific ELISA kit (Elabscience, Houston, TX, USA) according to the manufacturer’s instructions. Absorbance was read at 450 nm using a microplate reader (Multiskan Go, Thermo Fisher Scientific, Newington, NH, USA), and the values were expressed in pg/g tissue.

##### Liver Lipid Peroxidation

Liver MDA content was assessed by the TBARS assay, as described above. The absorbance of the final supernatant was read at 535 nm. MDA concentrations were calculated based on a standard curve and expressed as nmol/g protein, normalized to the total protein content in liver homogenates [20].

### 2.5. Statistical Analysis

Statistical analysis was conducted using IBM SPSS Statistics (Version 19; IBM Corp., Armonk, NY, USA). The normality of each variable within the experimental groups was assessed using the Shapiro–Wilk test. Parameters that exhibited normal distribution across all groups (e.g., ALT, TGF-β, plasma and tissue MDA) were analyzed using one-way analysis of variance (ANOVA). For these variables, Levene’s test was used to assess the homogeneity of variances. In cases where the assumption of homogeneity was met (e.g., TGF-β), Tukey’s HSD post hoc test was applied. When the variances were not homogeneous (e.g., ALT, plasma MDA, tissue MDA), Tamhane’s T2 post hoc test was used, considering that the group sample sizes were equal (n = 10 per group).

In contrast, for ordinal histological scores (e.g., necrosis, fibrosis, cellular infiltration), a Kruskal–Wallis test was first conducted to assess the overall group differences. Since significant differences were observed, pairwise comparisons were subsequently performed using the Mann–Whitney U test. Non-normally distributed continuous variables (e.g., syndecan-1) were also analyzed using the Kruskal–Wallis and Mann–Whitney U tests. Results for parametric data are reported as the mean ± standard error of the mean (SEM), and non-parametric data as the median (interquartile range (IQR)). A *p*-value < 0.05 was considered statistically significant. All graphical illustrations were generated using GraphPad Prism (Version 7; GraphPad Software, San Diego, CA, USA).

An a priori power analysis was conducted using G*Power (v3.1.9.7), based on previously published MTX-induced liver injury models [6,20,21]. These studies reported large treatment effects on oxidative stress and histopathological outcomes, with estimated effect sizes (Cohen’s *f*) ranging from 1.13 to 2.15. Accordingly, a sample size of 6–10 animals per group was sufficient to achieve 80% power at α = 0.05 (two-tailed). The group size used in this study (n = 10) was thus considered adequate to detect meaningful differences.

## 3. Results

### 3.1. Statistical Power and Sample Size Justification

Post hoc power analyses were conducted using G*Power (v3.1.9.7) to assess the adequacy of sample sizes across all endpoints. For non-parametric variables such as hepatocyte necrosis, fibrosis, cellular infiltration, and plasma syndecan-1 levels, large to very large effect sizes were observed (Cohen’s d = 1.19–3.87), with achieved powers of 95.2–97.7%. Similarly, for parametric variables analyzed by one-way ANOVA—including plasma and hepatic MDA, ALT, and TGF-β—very large effect sizes were noted (Cohen’s *f* = 1.64–1.89), with statistical power ranging from 97.4% to 99.5%. These results confirm that the sample sizes (typically n = 10 per group) were sufficient to detect meaningful differences.

### 3.2. Histopathological Scores

#### 3.2.1. Hepatocyte Necrosis

**Figure 2:**
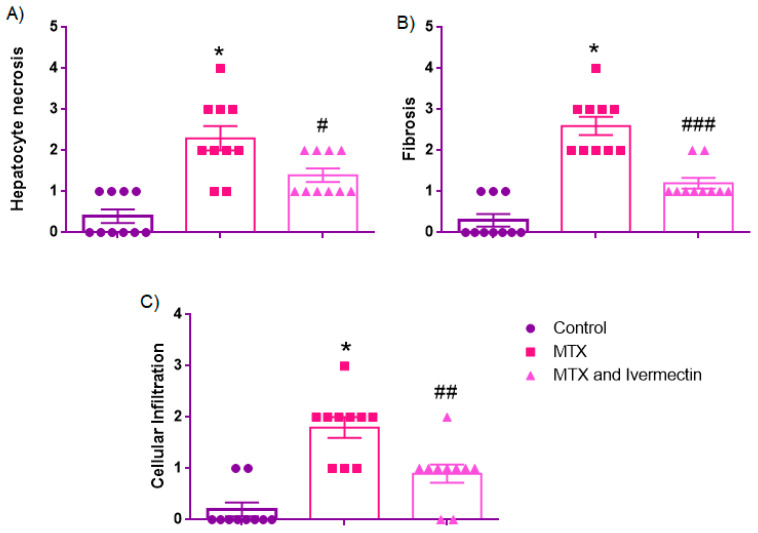
Histopathological scoring of liver injury: Hepatocyte necrosis (**A**), fibrosis (**B**), and cellular infiltration (**C**) scores in the control, methotrexate (MTX), and MTX + ivermectin groups. MTX administration significantly increased all histological injury scores, indicating extensive hepatocellular damage (* *p* < 0.001 vs. control group). Ivermectin treatment significantly attenuated necrosis (# *p* < 0.05), fibrosis (### *p* < 0.001), and cellular infiltration (## *p* < 0.01) compared to the MTX group. Data are presented as the median [interquartile range, IQR] due to the non-normal distribution of variables. Statistical comparisons were performed using the Mann–Whitney U test.

Hepatocyte necrosis scores differed significantly across the groups. The control group exhibited minimal necrosis, with a median score of 0.0 [IQR: 0.0–1.0], consistent with preserved hepatic architecture. MTX administration markedly increased the severity of necrosis (2.0 [1.75–3.0]), indicating widespread hepatocellular damage, including bridging necrosis. Ivermectin treatment significantly ameliorated this effect (1.0 [1.0–2.0]), reflecting partial histological protection. Statistical comparisons using the Mann–Whitney U test revealed significant differences between the control and MTX groups (*p* < 0.001), as well as between the MTX and MTX + ivermectin groups (*p* = 0.035). (Figure 2).

**Figure 3:**
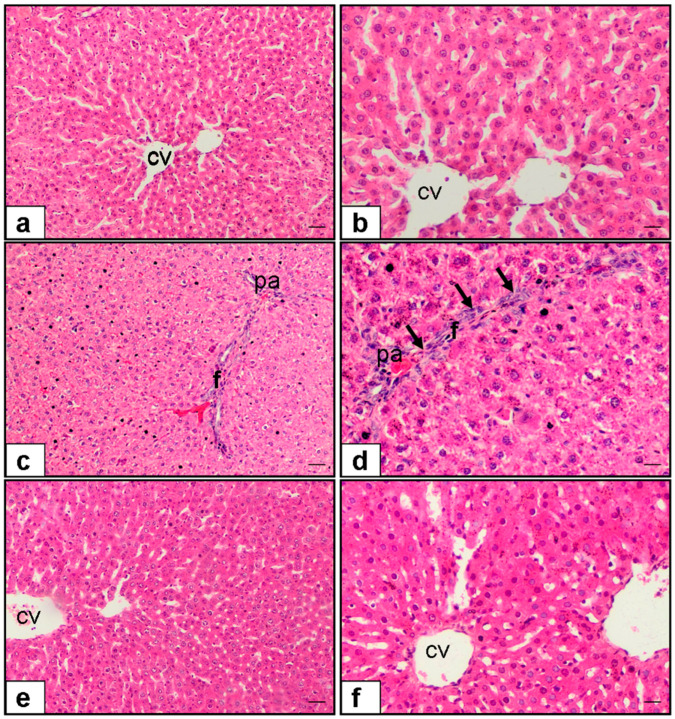
Representative histopathological images of liver tissue stained with hematoxylin and eosin (H&E) at 10× and 20× magnifications: (**a**,**b**) Control group: normal hepatic architecture with clearly visible central vein (CV). (**c**,**d**) Methotrexate (MTX) + saline group: marked bridging necrosis, portal fibrosis (F), and cellular infiltration (arrow) in the portal area (PA). (**e**,**f**) MTX + ivermectin group: reduced bridging necrosis, fibrosis, and inflammatory cell infiltration compared to the MTX group.

As illustrated in Figure 3, control liver sections (Figure 3a,b) maintained their structural integrity, with centrally located veins and no necrotic regions. MTX + saline-treated rats (Figure 3c,d) displayed widespread necrosis bridging across lobules, while ivermectin-treated rats (Figure 3e,f) showed substantially fewer necrotic zones (Figure 2 and Figure 3, and Table 1).

#### 3.2.2. Fibrosis

The fibrosis scores significantly varied across groups. The control group demonstrated minimal fibrotic changes (0.0 [0.0–1.0]), while MTX administration resulted in pronounced periportal and bridging fibrosis (2.5 [2.0–3.0]). Ivermectin treatment substantially reduced the severity of fibrosis (1.0 [1.0–1.25]), suggesting its antifibrotic effect. The Mann–Whitney U test indicated significant differences between the control and MTX groups (*p* < 0.001), and between the MTX and MTX + ivermectin groups (*p* < 0.001).

In Figure 3, the MTX + saline sections (Figure 3c,d) clearly demonstrate eosinophilic fibrotic bands within portal and periportal regions, while ivermectin-treated livers (Figure 3e,f) show reduced fibrosis and improved tissue architecture (Figure 2 and Figure 3, and Table 1).

#### 3.2.3. Cellular Infiltration

Inflammatory cell infiltration was nearly absent in the control group (0.0 [0.0–0.25]). MTX exposure markedly increased leukocytic infiltration (2.0 [1.0–2.0]), consistent with acute hepatic inflammation. Ivermectin treatment significantly mitigated infiltration (1.0 [0.75–1.0]), suggesting anti-inflammatory activity. The Mann–Whitney U test confirmed significant differences between the control and MTX groups (*p* < 0.001), and between the MTX and MTX + ivermectin groups (*p* = 0.009).

In Figure 3, MTX-exposed livers (Figure 3c,d) are marked by dense cellular infiltration, whereas Ivermectin-treated tissues (Figure 3e,f) reveal sparse inflammatory cell presence (Figure 2 and Figure 3, and Table 1).

### 3.3. Biochemical Parameters

#### 3.3.1. Liver TGF-β Levels (pg/g)

Statistical analysis revealed a significant difference in liver TGF-β levels among the groups (F(2,27) = 41.530, *p* < 0.001, one-way ANOVA). Levene’s test confirmed the homogeneity of variances (*p* = 0.832), and Tukey’s HSD post hoc test was applied. TGF-β levels were significantly higher in the MTX group (1.50 ± 0.05 pg/g) than in the control group (0.79 ± 0.05 pg/g, *p* < 0.001). Ivermectin treatment significantly reduced the TGF-β levels (1.05 ± 0.05 pg/g) compared to MTX (*p* < 0.001), indicating antifibrotic efficacy through downregulation of TGF-β signaling.

#### 3.3.2. Syndecan-1 (SDC-1)

**Figure 4:**
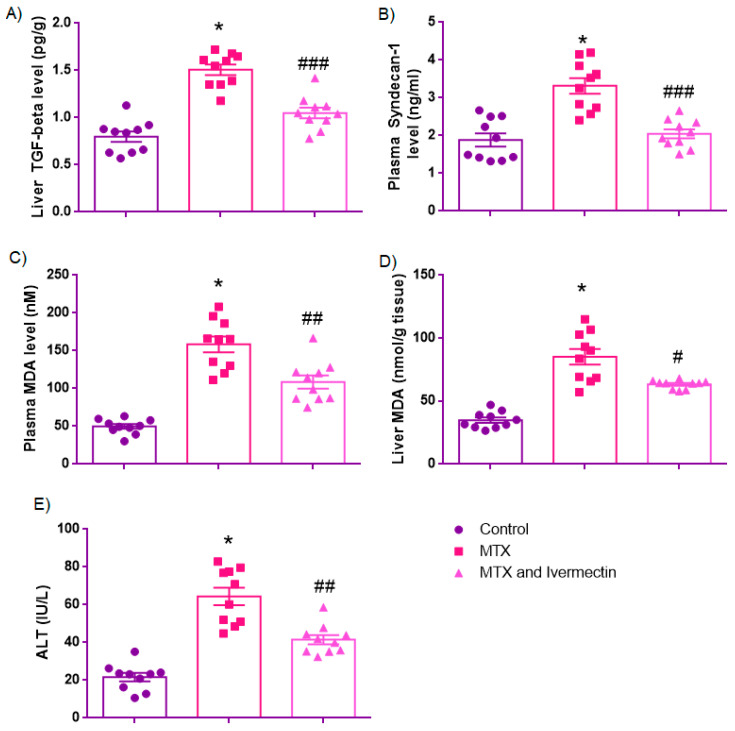
Effects of methotrexate (MTX) and ivermectin on biochemical markers of liver injury: Levels of hepatic transforming growth factor-beta (TGF-β) (**A**), plasma syndecan-1 (SDC1) (**B**), plasma malondialdehyde (MDA) (**C**), hepatic MDA (**D**), and alanine aminotransferase (ALT) (**E**) in the control, MTX, and MTX + ivermectin groups. MTX administration significantly increased all parameters compared to the control group (* *p* < 0.001, # *p* < 0.05, ## *p* < 0.01, ### *p* < 0.001 vs. methotrexate (MTX) group), indicating enhanced fibrogenesis, endothelial injury, oxidative stress, and hepatocellular damage. Ivermectin treatment significantly reduced these elevations, suggesting antifibrotic, antioxidant, and hepatoprotective effects.

The control group showed low plasma levels of syndecan-1 (1.71 [1.39–2.50] ng/mL), indicating intact endothelial glycocalyx. MTX administration significantly elevated SDC-1 levels (3.39 [2.69–3.92] ng/mL), suggesting severe endothelial injury. Ivermectin treatment reduced SDC-1 levels (2.01 [1.75–2.37] ng/mL), consistent with glycocalyx preservation. The Mann–Whitney U test revealed statistically significant differences both between the control and MTX groups (*p* < 0.001), and between the MTX and MTX + ivermectin groups (*p* < 0.001), (Figure 4, Table 2).

#### 3.3.3. Plasma MDA Levels (nM)

ANOVA showed a statistically significant difference in plasma MDA levels (F(2,27) = 45.736, *p* < 0.001). However, Levene’s test indicated unequal variances (*p* = 0.007), so Tamhane’s T2 post hoc test was used. MDA levels were significantly elevated in the MTX group (158.4 ± 10.45 nM) compared to the control group (49.66 ± 3.12 nM, *p* < 0.001). Ivermectin significantly reduced MDA levels (108.5 ± 8.68 nM) relative to MTX (*p* < 0.01; *p* = 0.005), demonstrating a protective antioxidant effect.

#### 3.3.4. Liver MDA Levels (nmol/g Tissue)

A significant difference in hepatic MDA levels was found among groups (F(2,27) = 43.482, *p* < 0.001). Levene’s test confirmed heterogeneity of variances (*p* < 0.001), so Tamhane’s T2 test was employed. MTX exposure increased liver MDA levels (85.21 ± 6.21 nmol/g) compared to the control group (34.80 ± 2.08 nmol/g, *p* < 0.001). Ivermectin significantly reduced hepatic MDA levels (63.18 ± 1.07 nmol/g) compared to MTX (*p* < 0.05; *p* = 0.018), supporting its role in mitigating oxidative membrane injury.

#### 3.3.5. ALT Levels (IU/L)

There was a significant overall difference in serum ALT levels (F(2,27) = 42.459, *p* < 0.001). Levene’s test showed unequal variances (*p* = 0.001); therefore, Tamhane’s T2 post hoc test was applied. ALT levels were significantly elevated in the MTX group (64.44 ± 4.63 IU/L) versus the control (21.61 ± 2.22 IU/L, *p* < 0.001), confirming hepatocellular damage. Ivermectin significantly reduced ALT levels (41.51 ± 2.45 IU/L) compared to MTX (*p* < 0.01, *p* = 0.002), indicating biochemical protection.

Data are presented as the mean ± standard error of the mean (SEM) for normally distributed variables, and as the median [interquartile range, IQR] for non-normally distributed variables (e.g., SDC1). Statistical analyses were performed using one-way ANOVA for parametric data and the Mann–Whitney U test for non-parametric data; # *p* < 0.05, ## *p* < 0.01, ### *p* < 0.001 vs. MTX group.

## 4. Discussion

In our study, MTX administration led to significant increases in MDA, TGF-β, and SDC1, reflecting oxidative stress, fibrogenesis, and liver injury. Notably, co-treatment with ivermectin significantly attenuated these effects, highlighting for the first time its potential hepatoprotective effects against MTX-induced liver damage, although the underlying mechanisms remain to be fully elucidated. To the best of our knowledge, this is the first report documenting ivermectin’s hepatoprotective effect in an MTX toxicity model. A novel aspect of this study is the simultaneous evaluation of syndecan-1 as a biomarker of liver injury and fibrosis in this context.

Methotrexate (MTX), although widely used in oncology and rheumatology, is frequently limited by hepatotoxic side effects. MTX-induced hepatotoxicity in humans generally occurs as a chronic, cumulative response to prolonged low-dose therapy, particularly in the context of rheumatoid arthritis or psoriasis [22,23]. However, high-dose MTX regimens used in chemotherapy have also been shown to cause acute liver injury, typically presenting with a rapid elevation in serum transaminases and other biochemical markers of hepatocellular damage [24,25,26]. Reflecting this clinical toxicity profile, several preclinical studies have adopted a single high-dose MTX administration (20 mg/kg, i.p.) in rodents to induce rapid-onset hepatic injury, which reliably reproduces oxidative stress, mitochondrial dysfunction, fibrosis, and apoptosis [27,28,29]. Despite this methodological divergence, the central pathogenic mechanism underlying both acute and chronic MTX-induced hepatotoxicity remains conserved. Specifically, ROS-mediated hepatocyte injury and necroinflammatory responses are implicated across both clinical and experimental settings [30,31]. Numerous antioxidant compounds, including N-acetylcysteine, curcumin, and resveratrol [32,33,34], as well as plant-derived agents such as pomegranate extract, berberine, and rhein [35,36,37], have previously demonstrated efficacy in mitigating MTX-induced hepatic injury by modulating oxidative stress, inflammation, and apoptotic pathways, notably through mechanisms involving the Nrf2 and NF-κB signaling cascades. These findings further validate the use of this experimental model for evaluating hepatoprotective candidates, as demonstrated by the significant attenuation of oxidative damage and fibrosis in the ivermectin co-treatment group.

A central mechanism underlying MTX-induced hepatotoxicity is oxidative stress [2,3]. In our study, MTX administration resulted in a substantial increase in malondialdehyde (MDA) levels in both plasma and liver tissue, indicating severe lipid peroxidation due to excessive reactive oxygen species (ROS). This biochemical evidence was accompanied by elevated serum ALT levels, reflecting hepatocyte membrane damage and loss of cellular integrity. These findings are in line with those of earlier studies reporting that high-dose MTX leads to ROS overproduction, oxidative membrane injury, and transaminase leakage [2,3,21]. Histologically, MTX-treated livers exhibited centrilobular necrosis, hepatocyte dropout, and dense inflammatory cell infiltration.

Importantly, ivermectin treatment markedly attenuated oxidative stress, as demonstrated by a 30–40% reduction in MDA levels in both plasma and liver tissue. Although ivermectin is not traditionally classified as an antioxidant, the observed effect implies an indirect contribution to redox homeostasis, potentially through FXR or Akt/mTOR pathways [13,16]. While these pathways were not directly examined in the current study, the histological findings are consistent with the possibility that ivermectin may confer protection against ROS-mediated hepatic injury. Similar to the findings of Ying et al. [38], who showed that ivermectin attenuated oxidative damage in a CCl_4_-induced liver fibrosis model, our study uniquely extends these observations to an acute MTX toxicity setting. This reduction in ALT and MDA aligns with our histological findings of less necrosis and supports the conclusion that ivermectin attenuates MTX-induced hepatocyte lysis. Collectively, our findings suggest that ivermectin may attenuate the MTX-induced oxidative stress and inflammatory responses, significantly preserving hepatocellular membrane integrity.

Additionally, MTX triggered a pronounced inflammatory response by stimulating ROS production, which activates hepatic immune cells such as Kupffer cells. Previous studies have shown that this activation leads to increased release of pro-inflammatory cytokines, including TNF-α and IL-6 [2,3]. In line with these findings, our histological evaluation revealed extensive inflammatory cell infiltration and bridging fibrosis, particularly in the portal areas, in the MTX group. Conversely, ivermectin treatment significantly mitigated hepatic inflammation. Inflammation scores were substantially lower in the MTX + ivermectin group, indicating reduced immune cell infiltration. Although cytokine levels were not directly measured, the histological improvements observed suggest a pronounced anti-inflammatory effect. This aligns with previous reports showing that ivermectin reduces hepatic macrophage accumulation and pro-inflammatory cytokine levels, including TNF-α [38]. The anti-inflammatory action of ivermectin may involve inhibition of NF-κB signaling or upregulation of anti-inflammatory mediators, although further molecular analysis is required to confirm these pathways [39,40].

Beyond serving as a damage marker, MDA may also play an active role in the development of fibrosis. Transforming growth factor-beta 1 (TGF-β1) is a well-established central mediator of hepatic fibrogenesis, primarily by inducing hepatic stellate cell (HSC) activation and subsequent collagen deposition [4,5,6]. Oxidative stress induced by MTX appears to facilitate this process through dual mechanisms: by activating Kupffer cells that release TGF-β, and by promoting the conversion of latent TGF-β to its active form via reactive oxygen species (ROS) [41]. Once activated, a feed-forward loop emerges, in which TGF-β further enhances ROS generation, perpetuating its own activation [3]. This self-amplifying cycle provides a mechanistic explanation for how elevated MDA levels, as indicators of ROS, drive fibrotic progression. In our study, MTX administration resulted in a twofold increase in hepatic TGF-β1 levels and a marked elevation in fibrosis scores (mean score: 2.5). Histopathological analysis confirmed the presence of early-stage bridging fibrosis, consistent with ROS-mediated HSC activation and extracellular matrix accumulation.

Crucially, treatment with ivermectin markedly attenuated this fibrogenic cascade. TGF-β1 levels in the MTX + ivermectin group were significantly lower (~1.1 pg/g vs. 1.5 pg/g in MTX alone), and the fibrosis score was reduced to ~1.1—nearly normalized. These observations raise the possibility that ivermectin may modulate TGF-β-mediated HSC activation and collagen production. Ying et al. [38] similarly demonstrated that ivermectin attenuated CCl₄-induced fibrosis by reducing α-SMA expression and hepatic collagen deposition [38]. In line with these findings, our results suggest that ivermectin may blunt TGF-β signaling and mitigate fibrotic progression in MTX-induced liver injury.

Syndecan-1 (SDC1), a heparan sulfate proteoglycan shed from hepatocytes and endothelial cells during tissue stress, serves as a sensitive indicator of endothelial injury and early fibrogenesis. Prior studies have demonstrated that TGF-β signaling in hepatocellular carcinoma induces MMP-7 and heparanase, promoting syndecan-1 shedding and enhancing fibrogenic signaling [42,43]. In contrast, syndecan-1 overexpression in transgenic mice delayed the onset of fibrosis, while conditioned media rich in shed syndecan-1 suppressed TGF-β1-induced myofibroblast activation by downregulating α-SMA and collagen I [43]. These findings suggest that syndecan-1 may act as a natural TGF-β antagonist during early injury. In our study, MTX exposure significantly elevated plasma SDC1 levels, indicating endothelial glycocalyx disruption and fibrotic activation. Ivermectin co-treatment markedly reduced SDC1 concentrations, suggesting attenuation of matrix remodeling and vascular damage.

This reduction likely stems from ivermectin’s capacity to alleviate upstream oxidative and inflammatory insults, thereby preventing hepatic stellate cell (HSC) activation. With fewer dying hepatocytes and lower ROS levels, less TGF-β is released, weakening the fibrogenic drive. Ivermectin might also modulate HSC-related signaling pathways, such as TGF-β/SMAD, or influence matrix turnover, although these hypotheses require direct validation. While its exact antifibrotic mechanism remains to be fully clarified, our findings support the hypothesis that ivermectin can substantially suppress MTX-induced liver fibrosis within a short timeframe. Its capacity to improve both biochemical markers (e.g., TGF-β, ALT) and histopathological scores supports the idea that ivermectin exerts disease-modifying rather than merely symptomatic effects, although further molecular studies are warranted.

## 5. Conclusions

**Figure 5:**
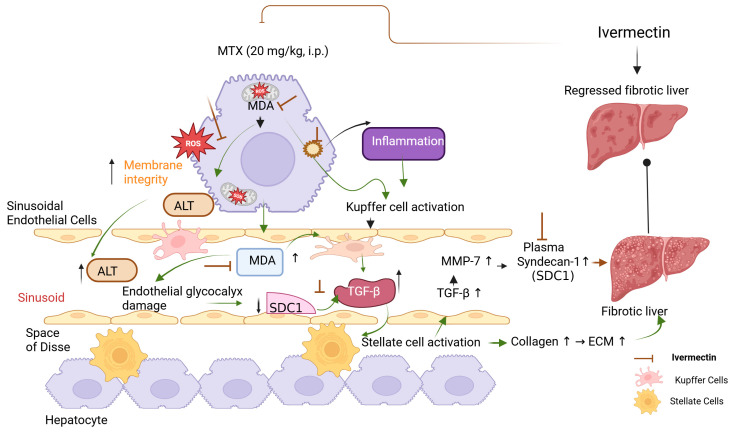
Graphical abstract. Abbreviations: MTX (methotrexate) was administered intraperitoneally (i.p.) and is known to induce liver injury through oxidative stress and inflammation. MDA (malondialdehyde) is a key marker of lipid peroxidation, while ROS (reactive oxygen species) represent upstream mediators of oxidative damage. ALT (alanine aminotransferase) is used as a serum biomarker of hepatocellular injury. SDC1 (syndecan-1) is a component of the endothelial glycocalyx that reflects vascular damage when elevated in plasma. TGF-β (transforming growth factor-beta) is a central fibrogenic cytokine, and MMP-7 (matrix metalloproteinase-7) is involved in extracellular matrix (ECM) remodeling during fibrotic progression.

Our findings suggest a tightly interlinked network in MTX-induced liver injury: ROS accumulation initiates TGF-β activation and SDC1 shedding; TGF-β, in turn, sustains ROS production and promotes fibrogenesis; and SDC1 may transiently buffer this response before being downregulated. This feed-forward cycle (MDA ↑ → TGF-β ↑ → HSC activation ↑ → plasma SDC1 ↑) supports the rationale for therapies that simultaneously target oxidative stress, inflammation, and fibrotic signaling. These preliminary findings support the hypothesis that ivermectin may interrupt this pathological loop by reducing oxidative stress, suppressing TGF-β activation, and preserving SDC1 levels, thereby attenuating the progression of hepatotoxicity and fibrosis. However, given the acute and limited nature of the experimental model, further pharmacokinetic, safety, and mechanistic studies are warranted before any clinical translation or therapeutic repurposing can be reasonably considered (Figure 5).

### Limitations

A limitation of the present study is the absence of an ivermectin-only group, which would have allowed for the evaluation of baseline histological and biochemical parameters independent of MTX-induced toxicity. However, previous studies have shown that ivermectin alone does not cause hepatic or systemic toxicity in healthy animals at comparable doses [44,45]. Future studies will include such a group to better delineate the intrinsic effects of ivermectin.

## Figures and Tables

**Table 1:** Histopathological parameters.

Parameters	Control	MTX	MTX and Ivermectin
Hepatocyte necrosis	0.0 [IQR: 0.0–1.0]	2.0 [1.75–3.0] *	1.0 [1.0–2.0] #
Fibrosis	0.0 [0.0–1.0])	2.5 [2.0–3.0] *	1.0 [1.0–1.25] ###
Cellular infiltration	0.0 [0.0–0.25]	2.0 [1.0–2.0] *	1.0 [0.75–1.0] ##

Data are expressed as the median [interquartile range, IQR]. Due to non-normal distribution, statistical comparisons were conducted using the Mann–Whitney U test; * *p* < 0.001 vs. control group; # *p* < 0.05, ## *p* < 0.01, ### *p* < 0.001 vs. methotrexate (MTX) group.

**Table 2:** Biochemical parameters.

Parameters	Control	MTX	MTX and Ivermectin
Liver TGF beta level (pg/g)	0.79 ± 0.05	1.50 ± 0.05 *	1.05 ± 0.05 ###
Plasma syndecan-1 level (ng/mL)	1.71 [1.39–2.50]	3.39 [2.69–3.92] *	2.01 [1.75–2.37] ###
Plasma MDA level (nM)	49.66 ± 3.12	158.4± 10.45 *	108.5 ± 8.68 ##
Liver MDA level (nmol/g tissue)	34.80 ± 2.08	85.21 ± 6.21 *	63.18 ± 1.07 #
ALT (IU/L)	21.61 ± 2.22	64.44 ± 4.63 *	41.51 ± 2.45 ##

Data are presented as the mean ± standard error of the mean (SEM) for normally distributed variables, and as the median [interquartile range, IQR] for non-normally distributed variables (e.g., syndecan-1). Statistical analyses were performed using one-way ANOVA for parametric data and the Mann–Whitney U test for non-parametric data; * *p* < 0.001 vs. control group; # *p* < 0.05, ## *p* < 0.01, ### *p* < 0.001 vs. methotrexate (MTX) group.

## Data Availability

Data are available upon request due to ethical/privacy reasons.
